# The Influence of Opioids on Pupil Initial Diameter and Pupillary Dilation Velocity in ICU Patients

**DOI:** 10.1111/aas.70080

**Published:** 2025-06-23

**Authors:** Valdemar Oskar Ingemann Sørensen, Stine Uhrenholt, Jannik Stokholm, Thomas Christensen, Morten Heiberg Bestle

**Affiliations:** ^1^ Department of Anaesthesiology and Intensive Care Copenhagen University Hospital – North Zealand Hilleroed Denmark; ^2^ Department of Neurology Copenhagen University Hospital – North Zealand Hilleroed Denmark; ^3^ Department of Clinical Medicine University of Copenhagen Copenhagen Denmark

## Abstract

**Background:**

Pupillary light reflex assessment is a promising modality for assessing autonomic nervous system status, given that it is fast, noninvasive, and provides quantitative results. Opioid therapy influences the pupil and is frequently used in the intensive care unit (ICU). We investigated the effect of opioids on pupillary size and dilation velocity in the pupillary light reflex in critically ill patients.

**Methods:**

This is a sub‐study on 55 patients from a prospective observational study acutely admitted to an ICU. All patients had daily blood samples and pupillary light reflex measurements. Blood samples were analyzed for opioids using mass spectrometry. Linear mixed models were used to estimate the possible association for each detected opioid to pupillary size and dilation velocity in the pupillary light reflex.

**Results:**

The pupil size before a light stimulus was closely associated to the dilation velocity after the light stimulus. The increase in the dilation velocity was 0.2 mm/s per 1 mm increase in pupil diameter before the light stimulus, 95% confidence interval [0.2–0.3], *p* < 0.001. Presence of fentanyl was associated with a smaller pupil size and a slower pupillary dilation velocity.

**Conclusions:**

The presence of fentanyl in blood samples from a mixed ICU population is associated with a slower pupillary dilation velocity in a concentration‐dependent matter.

**Editorial Comment:**

This study assesses the relation of observed opioid levels and light reflex pupillary size speed of change in an ICU cohort where there can be multiple classes of drugs present which influence autonomic nerve system function. Findings for different opioids, though most specifically fentanyl, show that opioid plasma concentrations have clear association with slower pupillary dilation velocity with light reflex response.

## 1 Introduction

The pupillary dilation is under control of the autonomic nervous system and the dilatation velocity may be of clinical interest as a marker of autonomic function [1, 2, 3, 4, 5]. The pupillary constriction after a light stimulus is mediated through parasympathetic activation, while the dilation is mediated through parasympathetic inhibition and sympathetic activation [6]. Automated pupillometry can be used to assess different parameters of the pupillary light reflex (PLR) and has potential as a tool to assess the interplay between sympathetic and parasympathetic function [2]. The pupillary light reflex dilation velocity (PLR_dil.vel._), measured as the dilation velocity in millimeters per second (mm/s), can therefore be used as a marker of the sympathetic activation [7, 8, 9]. In the intensive care unit (ICU) pupillometry has also been used as a marker of increased intracranial pressure and the depth of analgesia and sedation [10, 11].

Opioid therapy is the backbone of pain relief in the ICU [12, 13, 14, 15] and the mostly used opioids are morphine, fentanyl, and oxycodone [13, 16, 17, 18, 19]. Under normal circumstances, the Edinger–Westphal nucleus (EWN) (accessory oculomotor nucleus in the brainstem) mediates excitatory inputs to the pupillary muscles controlling the size of the pupil. The EWN itself is regulated by a constantly balanced interplay between excitatory and inhibitory inputs. After opioid administration, the pupil responds with miosis due to excitation of the pupillary constrictor muscle [3, 20, 21]. The mechanism is thought to be an opioid block of inhibition of the EWN [22, 23, 24]. Several studies with small sample sizes have investigated the potential effect of opioids on several parameters of the pupillary light reflex with divergent outcomes [3, 20, 21, 23, 24, 25, 26, 27, 28, 29, 30, 31, 32].

There is no certain conclusion on the association between opioids and the PLR_dil.vel._ in a clinical ICU setting. The aim of this study was to investigate if the pupillary dilation velocity was altered in ICU patients receiving opioids versus ICU patients not receiving opioids [12, 13, 14, 15]. We hypothesized that both PLR_init.dia._ and PLR_dil.vel._ are reduced when opioids are present.

## 2 Methods

This is a sub‐study of an original study investigating different modalities, including pupillometry, to assess function of the autonomic nervous system [33]. The original study was a prospective observational cohort study performed in the ICU at Copenhagen University Hospital – North Zealand, Hilleroed, Denmark. The inclusion criteria were age ≥ 18 years and acute admission to the ICU. Exclusion criteria were permanent ineligibility, for example, dementia or mental retardation, inability to perform delirium assessments due to, for example, language barrier or coma, withdrawal of active treatment, brain death, pregnancy, non‐obtainable consent according to the Danish national regulations, compulsory treatment, alcohol withdrawal delirium (delirium tremens), or prone position ventilation. Demographic data from this study have been published previously in the original study [33].

Approval of this sub‐study was obtained in August 2020 from the regional Committee on Health Research Ethics with ID: 74974/H‐18030538. All patients or next‐of‐kin gave written informed consent.

From October 2018 to January 2020, 55 patients were included. All had blood samples collected during weekday mornings for analyzes of opioids, and measurements of PLR_init.dia._ and PLR_dil.vel._ were performed. Blood samples were analyzed for concentrations of the most commonly prescribed opioids [34] (morphine, fentanyl, oxycodone, and tramadol) and opioid metabolites (morphine‐6bDG, OD‐tramadol [the main metabolite of tramadol]). Samples were analyzed with an ultra‐high‐performance liquid chromatography tandem mass spectrometry confirmatory method using pneumatically assisted electrospray ionization (UPLC‐ESI‐MS/MS) [35].

Pupillary measurements were conducted by automated pupillometry (PLR‐3000 from NeurOptics), which automatically measured the pupil size before, during, and after a fixed light stimulus. Both eyes were recorded, beginning with the right eye. Limits were set according to normative pupillary data with a maximum dilation velocity of 15 mm/s [36] and pupil size with a minimum of 1.5 mm and a maximum of 9 mm [7].

All confidence intervals (CIs) were 95% CIs. *p*‐values below 0.05 were considered significant. As a result of rounding, some CIs reported may include zero, despite *p*‐values less than 0.05.

Demographic data were taken from the medical records. We collected data on sex, age, ICU length of stay, mechanical ventilation, alcohol and smoking habits, Sequential Organ Failure Assessment (SOFA) score, Simplified Acute Physiology Score third generation (SAPS 3), delirium, and septic shock. Organ dysfunction was assessed with the SOFA score at admission [37]. A SAPS 3 was calculated at ICU admission [38, 39, 40]. Septic shock was assessed according to Sepsis‐3 guidelines [41]. Patients requiring vasopressors to maintain a mean arterial pressure (MAP) ≥ 65 mmHg and a serum lactate ≥ 2 mmol/L despite fluid resuscitation were regarded as having septic shock. Patients were screened for delirium twice a day with the Confusion Assessment Method for the ICU (CAM‐ICU) [42, 43].

All statistical analyzes were performed with RStudio (version 2023.03.1, build 446) [44]; for the linear mixed models the lmerTest package [45] was used.

Student's *t*‐test was used for normally distributed data, while Wilcoxon rank sum test was used for data with a nonparametric distribution. Fisher's exact test was used for comparing categorical variables.

Linear mixed models were used to explore the possible association between changes in PLR_init.dia._ and PLR_dil.vel._ in relation to either the presence or absence of each opioid, or the concentration of each opioid. PLR_init.dia._ and PLR_dil.vel._ were in turn modeled to each opioid in regard to if the opioid was present or absent at any given measurement occasion. We also modeled in turn the concentration of each opioid when detected to the average change in PLR_init.dia._ and PLR_dil.vel._ For all models, side (i.e., right or left eye) and measurement day were set as random effects nested within patients.

## 3 Results

Time period of data collection was from October 2018 to January 2020.

Data from 55 patients were analyzed. Of these, 37 were males (67.3%) and the mean age was 67.9 years (SD = 13.3), see Table 1. A total of 154 blood samples were collected and analyzed for opioids, and 263 pupillometry measurements from both eyes were included in the analyses for possible associations between PLR_init.dia._ or PLR_dil.vel._ and the opioids. The opioids were detected in a variable frequency; for example, fentanyl was present in 74 of 154 samples and in 33 of 55 patients, whereas tramadol was present in 6 of 154 samples and in 3 of 55 patients, see Table 2. PLR_init.dia._ was closely associated to PLR_dil.vel_. One mm increase in PLR_init.dia._ was associated with an increase in PLR_dil.vel._ of 0.2 mm/s, 95% CI [0.2–0.3], *p* value < 0.001. The values of PLR_init.dia._ and PLR_dil.vel._ were relatively evenly distributed regarding whether an opioid was present or not at a specific measurement occasion (Figures S1 and S2). When tested in linear mixed models, the presence of fentanyl was associated to a smaller PLR_init.dia._ with an average difference in the pupillary diameter of −0.9 mm, CI [−1.2 to −0.6] and *p* value < 0.001. The presence of fentanyl was also associated to a slower PLR_dil.vel._ with an average difference in the pupillary dilation velocity of −0.2 mm/s, CI [−0.3 to −0.1] and *p* value < 0.001; however, when the presence of fentanyl was combined with PLR_init.dia._ both as fixed effects, there was no significant association between fentanyl and PLR_dil.vel_. The presence of any of the other opioids had no statistically significant associations to neither PLR_init.dia._ nor PLR_dil.vel._ (Table 3). The concentration of fentanyl was, however, associated with a slower PLR_dil.vel._ (see Table 4 and Figure S3), also when the fentanyl concentration was combined with PLR_init.dia._ both as fixed effects. This association was still significant when using only PLR data measured concomitantly with a biobank sample later positive for fentanyl (Table S1). For fentanyl, we also checked if there was an uneven distribution of patients positive for fentanyl at any time versus patients not positive for fentanyl in relation to several covariables; that is, sex, age, length of stay, being on mechanical ventilation at any point, known alcohol overuse, current tobacco smoking, first SOFA score, SAPS 3 score, and delirium or septic shock at any point. There were significantly more patients positive for fentanyl also on mechanical ventilation, being current tobacco smokers, and having higher first SOFA scores, see Table S2a. We furthermore tested the same covariables in linear mixed models with fentanyl concentrations and either initial pupillary diameter, pupillary dilation velocity, or both, and found that fentanyl concentrations were still associated with a slower pupillary dilatation velocity in models where the various covariables also had a significant association to the pupillary dilation velocity, see Table S2b.

**TABLE 1:** Demographics.

All patients, *n*	55
Male sex, *n* (%)	37 (67.3)
Age, mean (SD)	67.9 (13.4)
Length of stay (days), median (IQR)	8.9 (13.7)
Ventilator, *n* (%)	33 (60.0)
Alcohol^a^, *n* (%)	17 (34.0)
Current smoking^b^, *n* (%)	14 (28.0)
First SOFA, median (IQR)	6 (7)
SAPS 3, mean (SD)	70.6 (15.1)
Delirium (%)	22 (43.1)
Septic shock (%)	25 (45.5)

Abbreviations: IQR = interquartile range; *n* = number; SD = standard deviation.

^a^ Alcohol intake above recommended by Danish Health Authorities in 2020 (84 g of pure alcohol for women/168 g for men per week). Alcohol consumption data from 50 patients.

^b^ Smoking data from 50 patients.

**TABLE 2:** Overview of each opioid's presence.

	Patients, *n*	Measurements, *n*	Median^a^	IQR^a^	Minimum^a^	Maximum^a^
Fentanyl	31	74	1.0	1.6	0.1	12.0
Morphine	13	21	0.0053	0.0034	0.003	0.95
Morphine‐6bDG	19	37	0.027	0.041	0.0051	0.19
Oxycodone	12	17	0.0086	0.0202	0.003	0.061
Tramadol	3	6	0.0245	0.012	0.0083	0.3

*Note:* There was a total of 55 patients with 154 measurement occasions in total. Note that the median, IQR, minimum, and maximum values are only summarized from those samples with the specified opioid present.

Abbreviations: IQR = interquartile range; mg/kg = milligrams per kilogram; *n* = number.

^a^ Micrograms per kilogram for fentanyl and milligrams per kilogram for the other opioids.

**TABLE 3:** PLR initial diameter and dilation velocity in relation to opioids.

	Difference	Confidence interval	*p*
(A) PLR initial diameter (in mm)			
Fentanyl	−0.9	−1.2 to −0.6	< 0.001
Morphine	−0.1	−0.6 to 0.3	0.53
Morphine‐6bDG	−0.2	−0.5 to 0.2	0.38
Oxycodone	0	−0.6 to 0.5	0.87
Tramadol	−0.9	−1.8 to 0	0.050
(B) PLR dilation velocity (in mm/s)			
Fentanyl	−0.2	−0.3 to −0.1	< 0.001
Morphine	0	−0.2 to 0.1	0.93
Morphine‐6bDG	0	−0.1 to 0.1	0.90
Oxycodone	−0.1	−0.3 to 0	0.14
Tramadol	−0.2	−0.5 to 0.1	0.19
(C) PLR dilation velocity (in mm/s)			
PLR initial diameter	0.2	0.2 to 0.3	< 0.001
Fentanyl	0	−0.1 to 0.1	0.55
PLR initial diameter	0.2	0.2 to 0.3	< 0.001
Morphine	0	−0.1 to 0.1	0.59
PLR initial diameter	0.2	0.2 to 0.3	< 0.001
Morphine‐6bDG	0	−0.1 to 0.1	0.45
PLR initial diameter	0.2	0.2 to 0.3	< 0.001
Oxycodone	−0.1	−0.2 to 0	0.048
PLR initial diameter	0.2	0.2 to 0.3	< 0.001
Tramadol	0	−0.2 to 0.2	0.91

*Note:* The table shows different linear mixed models with each opioid in relation to the two variables “PLR initial diameter” and “PLR dilation velocity.” All models are based on all 55 patients and all 263 measurements. In section A, each opioid is modeled to PLR initial diameter in turn. The results shown are the average difference in PLR initial diameter in relation to the presence of each opioid, that is, the presence of fentanyl was significantly associated with an average difference in initial diameter of −0.9 mm (95% confidence interval −1.2 to −0.6 mm, *p* value < 0.001) for PLR measurements where fentanyl was present in the blood sample as compared to PLR measurements where fentanyl was absent in the blood sample. In section B, each opioid is modeled to PLR dilation velocity and in section C, each opioid is modeled again to PLR dilation velocity, but with PLR initial diameter as a covariable (fixed effect).

**TABLE 4:** PLR initial diameter and dilation velocity in relation to opioid concentrations.

All patients	Difference	Confidence interval	*p*	Patients	Observations
(A) PLR initial diameter (in mm)					
Fentanyl	−0.2	−0.3 to −0.1	< 0.001	55	263
Morphine	0.6	−1.1 to 2.3	0.48	55	263
Morphine‐6bDG	−2.5	−8.5 to 3.5	0.41	55	263
Oxycodone	−8.2	−27.6 to 11.4	0.41	55	263
Tramadol	−3.9	−10.0 to 2.1	0.20	55	263
(B) PLR dilation velocity (in mm/s)					
Fentanyl	−0.1	−0.1 to 0	< 0.001	55	263
Morphine	0.6	0.1 to 1.2	0.025	55	263
Morphine‐6bDG	−0.8	−2.8 to 1.1	0.40	55	263
Oxycodone	−7.3	−13.6 to −1.0	0.025	55	263
Tramadol	−0.9	−2.9 to 1.1	0.37	55	263
(C) PLR dilation velocity (in mm/s)					
PLR initial diameter	0.2	0.2 to 0.3	< 0.001	55	263
Fentanyl	0	−0.1 to 0	0.001		
PLR initial diameter	0.2	0.2 to 0.3	< 0.001	55	263
Morphine	0.5	0.1 to 0.8	0.010		
PLR initial diameter	0.2	0.2 to 0.3	< 0.001	55	263
Morphine‐6bDG	−0.2	−1.6 to 1.1	0.73		
PLR initial diameter	0.2	0.2 to 0.3	< 0.001	55	263
Oxycodone	−5.4	−9.7 to −1.2	0.014		
PLR initial diameter	0.2	0.2 to 0.3	< 0.001	55	263
Tramadol	0	−1.3 to 1.3	0.98		

*Note:* The table shows different linear mixed models with each opioid in relation to the two variables “PLR initial diameter” and “PLR dilation velocity.” In section A, each opioid is modeled to PLR initial diameter in turn. The results shown are the average difference in PLR initial diameter in relation to the concentration of each opioid (in micrograms (μg) per kilogram for fentanyl and milligrams per kilogram for the other opioids), that is, 1 μg per kilogram of fentanyl was significantly associated with an average difference in initial diameter of −0.2 mm. In section B, each opioid is modeled to PLR dilation velocity and in section C, each opioid is modeled again to PLR dilation velocity, but with PLR initial diameter as a covariable.

## 4 Discussion

In this sub‐study in 55 ICU patients, we found that the presence of fentanyl was associated with a smaller PLR_init.dia._ and a lower PLR_dil.vel_. There was also an association between fentanyl in a concentration‐dependent manner and a slower PLR_dil.vel._

PLR_init.dia._ being smaller with the presence of fentanyl was expected based on previous investigations demonstrating that opioids cause pupillary constriction [3, 21, 23, 24, 32]. The concentration‐dependent association between a slower PLR_dil.vel._ and fentanyl is consistent with results from a previous study suggesting that fentanyl influences and depresses the reflex dilation [23].

PLR_init.dia._ was smaller with fentanyl present but not with the other opioids, which was opposite to what we expected. An explanation could be that our distribution of measurements with and without opioids was unequal for all other opioids than fentanyl. Another reason could be that the patients admitted to an ICU are suffering from severe diseases, which is known to activate the sympathetic nervous system and dilate the pupil [3]. Furthermore, fentanyl is often used as a continuous infusion for sedation and analgesia in the ICU, while the other investigated opioids are most commonly administered as bolus injections as required [13, 14, 16]. Finally, although the concentrations of opioids appear similar, fentanyl is 50–100 times more potent than morphine [15, 46]. This could explain the large differences for both PLR_init.dia._ and PLR_dil.vel._ (Table 4).

### 4.1 Strengths and Limitations

First, patients who received fentanyl were almost equally distributed across groups (131 observations with presence of opioids and 132 without) which makes results statistically strong and reliable.

Secondly, patients included in our study received standard care and therapy, thus making our study clinically relevant and comparable to everyday practice and clinical situations. In addition, a considerable strength is that this is, to our knowledge, the first study measuring opioid concentrations with such certainty by using a highly sensitive method, UPLC‐ESI‐MS/MS, and combining these with PLR measurements.

Our study also has limitations. For morphine, morphine‐6bDG, and oxycodone patients were unequally distributed across groups (40, 66, and 25 observations with presence of opioids) making statistical evidence less powerful, while the distribution for tramadol was largely unequal with only three patients (a total of 12 measurements) with tramadol present, and potentially unreliable. It was also a limitation that patients received standard treatment and baseline treatment, including medication that potentially could have influenced the pupillary measurements. This involves drugs with known sympathomimetic effects that theoretically could affect the PLR (through mechanisms mentioned in Section 1), for example, adrenaline, noradrenaline, β‐blocking drugs, different acting β2‐agonists, antidepressants, and antipsychotics. Standard treatment, for example in mechanically ventilated patients, includes sedatives with known effects on the PLR (e.g., benzodiazepines, propofol, haloperidol, dexmedetomidine) [22, 32, 47, 48, 49, 50, 51, 52, 53]. Additionally, during the inclusion period the Agents Intervening against Delirium in the Intensive Care Unit Trial (AID‐ICU) was running. This trial investigated the benefits and harms of haloperidol for the treatment of delirium in adult ICU patients [54]. Patients experiencing delirium were randomized and blinded to haloperidol or placebo during inclusion which potentially could influence pupillary measurements. It has previously been demonstrated that haloperidol can constrict the pupil and depress the PLR_dil.vel._ [55]. Furthermore, delirium itself has been suggested to influence the PLR_dil.vel._ [56]; however this association was not found in the original study [33]. We considered this a bias of unknown degree.

Unfortunately, we did not collect data on all medications administered, which restricted our ability to adjust for these influences during the statistical processing of the data.

Another limitation was that of the original 91 patients with PLR data, only 55 patients had biobank samples remaining for opioid analyses. Although the aim was to collect blood samples every weekday morning from all patients, this did not always happen. Moreover, some of the collected blood samples did not have enough material to send for the opioid analyses after other analyses made for the original study [33].

Before widespread use of pupillometers in the ICU, we propose further research on how and if any other common medications may be associated with changes is pupillary measurements. For better evidence large, randomized trials with equal distribution of measurements are needed to determine reliable results of the possible direct effect of opioids on the PLR_init.dia._ and PLR_dil.vel_.

## 5 Conclusion

The presence of fentanyl in blood samples from a mixed ICU population is associated with a slower pupillary dilation velocity in a concentration‐dependent manner.

## Author Contributions

The manuscript was drafted by V.O.I.S. The data were collected by S.U. Statistical analyses were performed by J.S. All authors contributed to the study design, discussion, and revision of the manuscript.

## Conflicts of Interest

The authors declare no conflicts of interest.

## Supporting information


**Figure S1.** Plots of PLR_init.dia._ in respect to each opioid.

**Figure S2.** Plots of PLR_dil.vel._ in respect to each opioid.

**Figure S3.** Plots of PLR_init.dia._ and PLR_dil.vel._ in respect to fentanyl concentrations.

**Table S1.** PLR initial diameter and dilation velocity in relation to opioid concentrations.

**Table S2.** (a) Fentanyl and covariables. (b) PLR initial diameter and dilation velocity in relation to fentanyl concentrations and covariables for all patients.

## Data Availability

Data sharing is not possible as data involve patient information and is confidential.
