# Supplementary figures and images for: The Influence of Opioids on Pupil Initial Diameter and Pupillary Dilation Velocity in ICU Patients
Source: Acta Anaesthesiol Scand. 2025 Jun 23;69(6):e70080. doi: 10.1111/aas.70080 (PMC12185176; doi:10.1111/aas.70080)

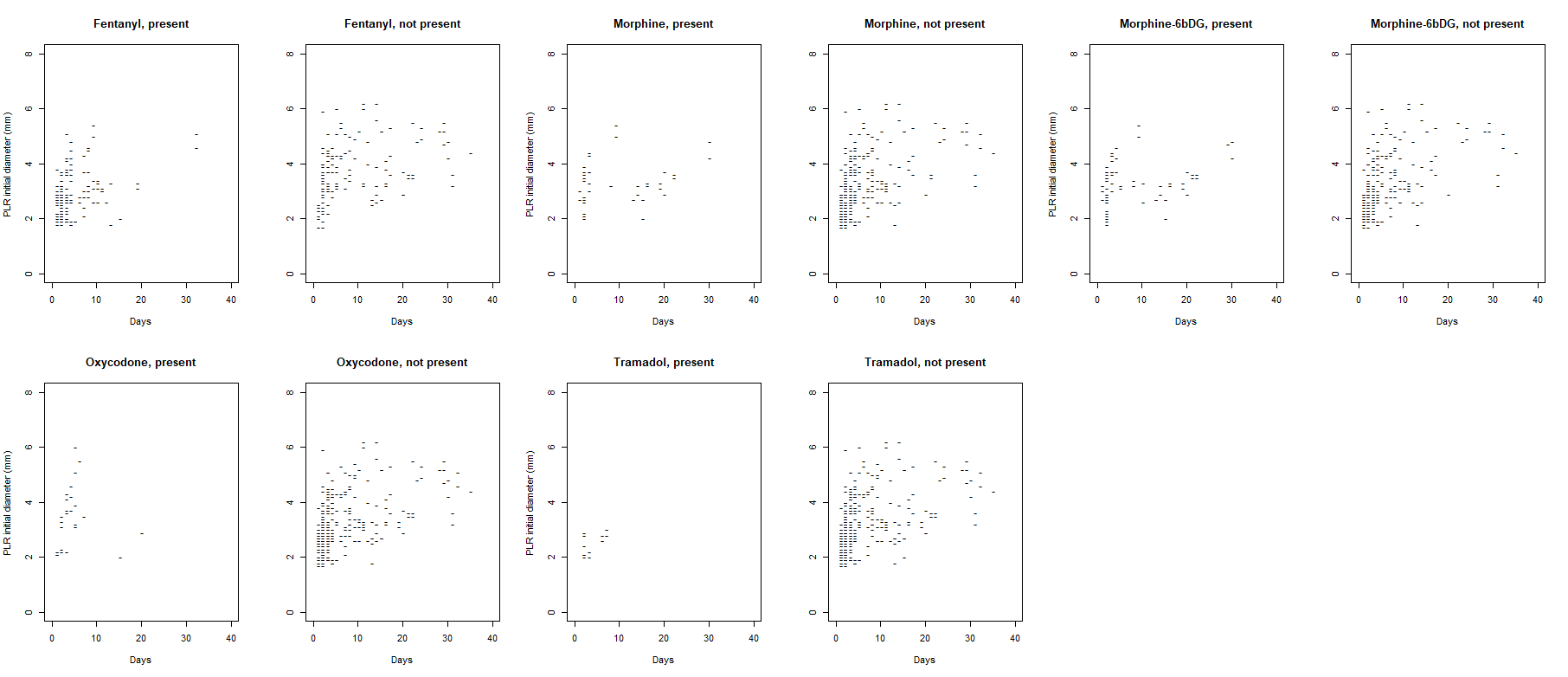


Supplemental figure 1. Plots of PLRinit.dia. in respect to each opioid.

Supplement: Supplementary file 1 — Figure S1. Plots of PLRinit.dia. in respect to each opioid. [file AAS-69-0-s002.docx]

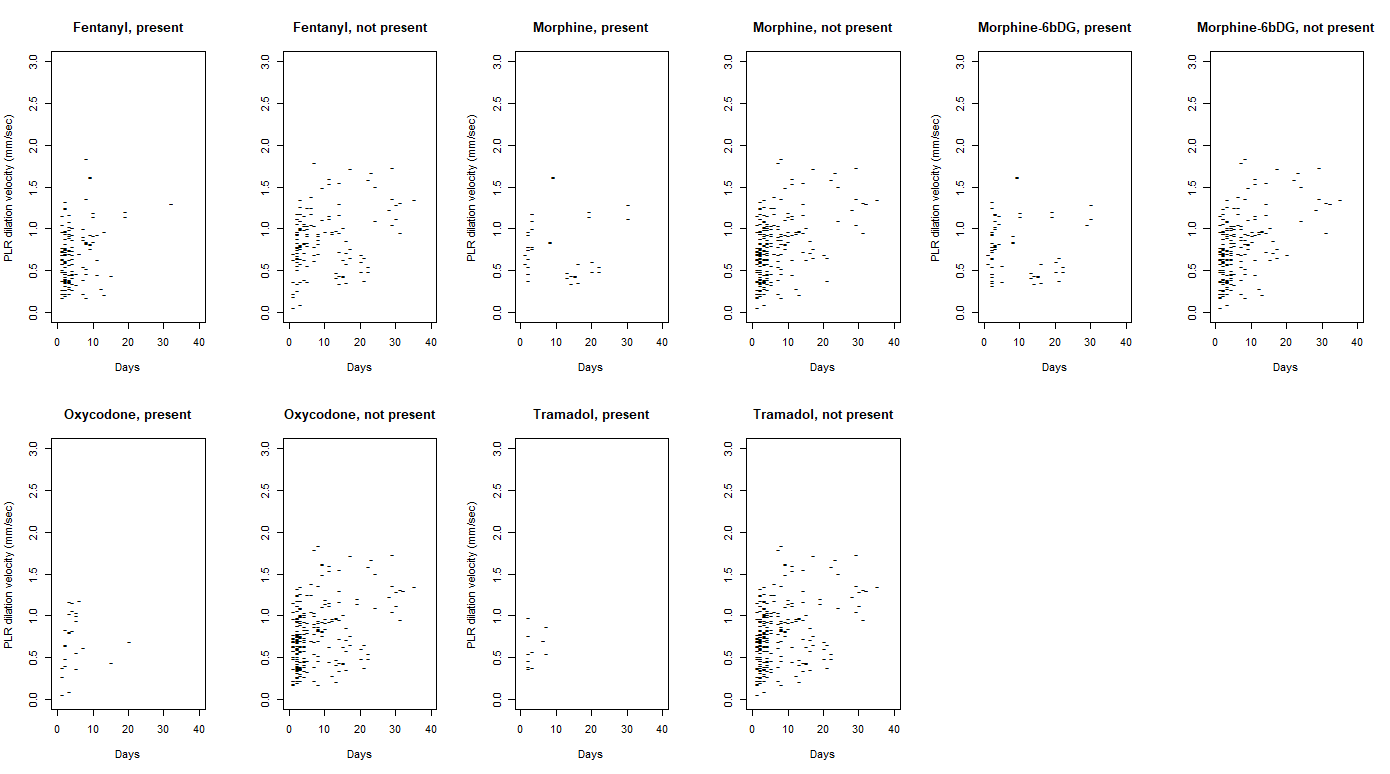


Supplemental figure 2. Plots of PLRdil.vel. in respect to each opioid.

Supplement: Supplementary file 2 — Figure S2. Plots of PLRdil.vel. in respect to each opioid. [file AAS-69-0-s004.docx]
