# Supplementary material for: The Influence of Opioids on Pupil Initial Diameter and Pupillary Dilation Velocity in ICU Patients
Source: Acta Anaesthesiol Scand. 2025 Jun 23;69(6):e70080. doi: 10.1111/aas.70080 (PMC12185176; doi:10.1111/aas.70080)
Supplement: Supplementary file 3 — Figure S3. Plots of PLRinit.dia. and PLRdil.vel. in respect to fentanyl concentrations. [file AAS-69-0-s003.docx]

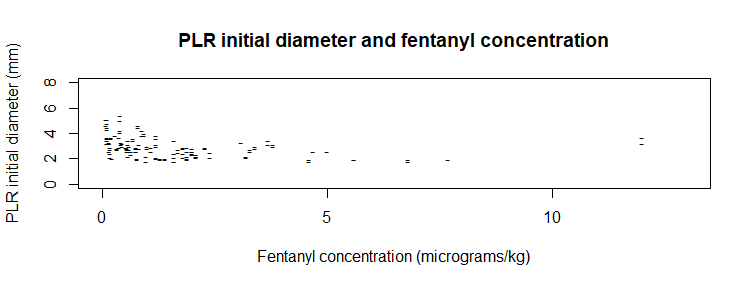


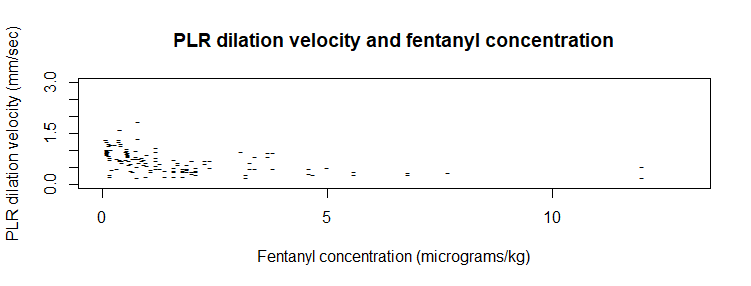


Supplemental figure 3. Plots of PLR_init.dia._ and PLR_dil.vel._ in respect to fentanyl concentrations.
