# Supplementary material for: The Influence of Opioids on Pupil Initial Diameter and Pupillary Dilation Velocity in ICU Patients
Source: Acta Anaesthesiol Scand. 2025 Jun 23;69(6):e70080. doi: 10.1111/aas.70080 (PMC12185176; doi:10.1111/aas.70080)
Supplement: Supplementary file 4 — Table S1. PLR initial diameter and dilation velocity in relation to opioid concentrations. [file AAS-69-0-s005.docx]

| Supplemental table 1. PLR initial diameter and dilation velocity in relation to opioid concentrations. | | | | | |  |
| --- | --- | --- | --- | --- | --- | --- |
| A | |  | | |  |  |
| Subselection | | PLR initial diameter (in millimeters) | | |  |  |
|  | | Difference | Confidence interval | P value | Patients | Observations |
| Fentanyl | | -0.1 | -0.2 to 0 | 0.021 | 31 | 132 |
| Morphine | | 0 | -1.7 to 1.7 | 0.99 | 13 | 40 |
| Morphine-6bDG | | -3.9 | -10.3 to 2.4 | 0.21 | 19 | 66 |
| Oxycodone | | -16.9 | -31.8 to -2.6 | 0.060 | 12 | 25 |
| Tramadol | | -2.7 | -4.0 to -1.2 | 0.038 | 3 | 12 |
|  | | | | |  |  |
| B | |  | | |  |  |
| Subselection | | PLR dilation velocity (in millimeters per second) | | | |  |
|  | | Difference | Confidence interval | P value | Patients | Observations |
| Fentanyl | | -0.1 | -0.1 to 0 | <0.001 | 31 | 132 |
| Morphine | | 0.4 | -0.2 to 1.1 | 0.22 | 13 | 40 |
| Morphine-6bDG | | -2.9 | -5.0 to -0.9 | 0.007 | 19 | 66 |
| Oxycodone | | -10.6 | -14.9 to -5.9 | 0.003 | 12 | 25 |
| Tramadol | | -0.8 | -1.5 to 0 | 0.069 | 3 | 12 |
|  | | | | |  |  |
| C | |  | | |  |  |
| Subselection | | PLR dilation velocity (in millimeters per second) | | | |  |
|  | | Difference | Confidence interval | P value | Patients | Observations |
| PLR initial diameter | | 0.3 | 0.2 to 0.3 | <0.001 | 31 | 132 |
| Fentanyl | | -0.1 | -0.1 to 0 | <0.001 |  |  |
|  | |  |  |  |  |  |
| PLR initial diameter | | 0.3 | 0.2 to 0.4 | <0.001 | 13 | 40 |
| Morphine | | 0.4 | 0 to 0.8 | 0.054 |  |  |
|  | |  |  |  |  |  |
| PLR initial diameter | | 0.3 | 0.2 to 0.4 | <0.001 | 19 | 66 |
| Morphine-6bDG | | -1.2 | -3.7 to 0.9 | 0.21 |  |  |
|  | |  |  |  |  |  |
| PLR initial diameter | | 0.2 | 0.1 to 0.3 | <0.001 | 12 | 25 |
| Oxycodone | | -6.3 | -9.8 to -1.0 | 0.013 |  |  |
|  | |  |  |  |  |  |
| PLR initial diameter | | 0.5 | 0 to 0.7 | 0.033 | 19 | 66 |
| Tramadol | | 0.4 | -0.7 to 1.3 | 0.47 |  |  |

The table shows different linear mixed models with each opioid in relation to the two variables "PLR initial diameter" and "PLR dilation velocity". The three sections A, B and C are repeats of the same sections in Table 4 with the exception that only PLR measurements where each opioid detected in a blood sample was used. In section A, each opioid is modelled to PLR initial diameter in turn. The results shown are the average difference in PLR initial diameter in relation to the concentration of each opioid (in micrograms per kilogram for fentanyl and in milligrams per kilogram for the other opioids). In section B, each opioid is modelled to PLR dilation velocity and in section C, each opioid is modelled again to PLR dilation velocity, but with PLR initial diameter as a covariable.
