# Supplementary material for: The Influence of Opioids on Pupil Initial Diameter and Pupillary Dilation Velocity in ICU Patients
Source: Acta Anaesthesiol Scand. 2025 Jun 23;69(6):e70080. doi: 10.1111/aas.70080 (PMC12185176; doi:10.1111/aas.70080)
Supplement: Supplementary file 5 — Table S2. (a) Fentanyl and covariables. (b) PLR initial diameter and dilation velocity in relation to fentanyl concentrations and covariables for all patients. [file AAS-69-0-s001.docx]

| Supplemental table 2a. Fentanyl and covariables. | | | | | |
| --- | --- | --- | --- | --- | --- |
| Covariable | Fentanyl, present | Fentanyl,  not present | Difference | Confidence interval | P value |
| Male sex, *n (%)* | 19 (61.3) | 18 (75.0) |  |  | 0.39 |
| Age, *mean (SD)* | 65.5 (13.6) | 71.0 (12.7) | -5.5 | -12.7 to 1.8 | 0.14 |
| Length of stay (days), *median (IQR)* | 9.5 (14.5) | 8 (10.6) | 2.9 | -1.1 to 6.2 | 0.18 |
| Ventilator, *n (%)* | 24 (77.4) | 9 (37.5) |  |  | 0.005 |
| Alcohol*, *n (%)* | 10 (37.0) | 7 (30.4) |  |  | 0.77 |
| Current smoking**, *n (%)* | 12 (42.9) | 2 (9.1) |  |  | 0.011 |
| First SOFA, *median (IQR)* | 9 (5) | 4 (5) | 4 | 1 to 5 | 0.005 |
| SAPS 3, *mean (SD)* | 71.5 (16.4) | 69.5 (13.4) | 2.0 | -6.3 to 10.3 | 0.63 |
| Delirium***, *n (%)* | 13 (48.1) | 9 (37.5) |  |  | 0.57 |
| Septic shock***, *n (%)* | 17 (54.8) | 8 (33.3) |  |  | 0.17 |

Fentanyl, present / Fentanyl, not present denotes whether or not fentanyl was detected in a given patient’s blood samples at any point.

IQR = interquartile range, SD = standard deviation.

*) Alcohol intake above recommended by Danish Health Authorities in 2020 (84g of pure alcohol for women/168g for men per week). Alcohol consumption data from 50 patients.

**) Smoking data from 50 patients

***) If in septic shock/delirium at some point throughout the ICU stay.

| Supplemental table 2b. PLR initial diameter and dilation velocity in relation to fentanyl concentrations and covariables for all patients. | | | |
| --- | --- | --- | --- |
| A | PLR initial diameter (in millimeters) | | |
|  | Difference | Confidence interval | P value |
| Fentanyl | -0.2 | -0.3 to -0.1 | <0.001 |
| Male sex | 0.1 | -0.3 to 0.6 | 0.57 |
|  |  |  |  |
| Fentanyl | -0.2 | -0.3 to -0.1 | <0.001 |
| Age | 0 | 0 to 0 | 0.92 |
|  |  |  |  |
| Fentanyl | -0.2 | -0.3 to -0.1 | <0.001 |
| Length of stay | 0 | 0 to 0 | 0.003 |
|  |  |  |  |
| Fentanyl | -0.1 | -0.2 to 0 | 0.005 |
| Ventilator | -0.3 | -0.7 to 0 | 0.053 |
|  |  |  |  |
| Fentanyl | -0.2 | -0.2 to -0.1 | 0.002 |
| Alcohol* | 0 | -0.4 to 0.4 | 1 |
|  |  |  |  |
| Fentanyl | -0.2 | -0.3 to -0.1 | <0.001 |
| Current smoking** | -0.3 | -0.7 to 0.2 | 0.24 |
|  |  |  |  |
| Fentanyl | -0.1 | -0.2 to 0 | 0.16 |
| SOFA | -0.1 | -0.1 to -0.1 | <0.001 |
|  |  |  |  |
| Fentanyl | -0.2 | -0.3 to -0.1 | <0.001 |
| SAPS 3 | 0 | 0 to 0 | 0.24 |
|  |  |  |  |
| Fentanyl | 0 | -0.1 to 0.2 | 0.53 |
| Delirium*** | 0.1 | -0.3 to 0.5 | 0.55 |
|  |  |  |  |
| Fentanyl | -0.1 | -0.2 to 0 | 0.079 |
| Septic shock*** | -0.8 | -1.1 to -0.4 | <0.001 |
|  | | | |
| B | PLR dilation velocity (in millimeters per second) | | |
|  | Difference | Confidence interval | P value |
| Fentanyl | -0.1 | -0.1 to 0 | <0.001 |
| Male sex | 0.1 | -0.1 to 0.2 | 0.52 |
|  |  |  |  |
| Fentanyl | -0.1 | -0.1 to 0 | <0.001 |
| Age | 0 | 0 to 0 | 0.86 |
|  |  |  |  |
| Fentanyl | -0.1 | -1.1 to -0.1 | <0.001 |
| Length of stay | 0 | 6.6 to 0 | 0.053 |
|  |  |  |  |
| Fentanyl | -0.1 | -0.1 to 0 | <0.001 |
| Ventilator | -0.1 | -0.2 to 0 | 0.01 |
|  |  |  |  |
| Fentanyl | -0.1 | -0.1 to 0 | <0.001 |
| Alcohol* | 0 | -0.1 to 0.2 | 0.77 |
|  |  |  |  |
| Fentanyl | -0.1 | -0.1 to -0.1 | <0.001 |
| Current smoking** | 0 | -0.1 to 0.2 | 0.64 |
|  |  |  |  |
| Fentanyl | 0 | -0.1 to 0 | 0.017 |
| SOFA | 0 | -0.1 to 0 | <0.001 |
|  |  |  |  |
| Fentanyl | -0.1 | -0.1 to 0 | <0.001 |
| SAPS 3 | 0 | 0 to 0 | 0.52 |
|  |  |  |  |
| Fentanyl | 0 | -0.1 to 0 | 0.25 |
| Delirium*** | 0.1 | -0.1 to 0.2 | 0.30 |
|  |  |  |  |
| Fentanyl | -0.1 | -0.1 to 0 | 0.003 |
| Septic shock*** | -0.2 | -0.4 to -0.1 | <0.001 |
|  | | | |
| C | PLR dilation velocity (in millimeters per second) | | |
|  | Difference | Confidence interval | P value |
| PLR initial diameter | 0.2 | 0.2 to 0.3 | <0.001 |
| Fentanyl | 0 | -0.1 to 0 | 0.001 |
| Male sex | 0 | -0.1 to 0.1 | 0.76 |
|  |  |  |  |
| PLR initial diameter | 0.2 | 0.2 to 0.3 | <0.001 |
| Fentanyl | 0 | -0.1 to 0 | 0.001 |
| Age | 0 | 0 to 0 | 0.93 |
|  |  |  |  |
| PLR initial diameter | 0.2 | 0.2 to 0.3 | <0.001 |
| Fentanyl | 0 | -0.1 to 0 | 0.001 |
| Length of stay | 0 | 0 to 0 | 0.88 |
|  |  |  |  |
| PLR initial diameter | 0.2 | 0.2 to 0.2 | <0.001 |
| Fentanyl | 0 | -5.4 to 0 | 0.008 |
| Ventilator | -0.1 | -1.4 to 0 | 0.079 |
|  |  |  |  |
| PLR initial diameter | 0.2 | -0.2 to 0.2 | <0.001 |
| Fentanyl | 0 | -0.1 to 0 | 0.002 |
| Alcohol* | 0 | -0.1 to 0.1 | 0.67 |
|  |  |  |  |
| PLR initial diameter | 0.2 | 0.2 to 0.2 | <0.001 |
| Fentanyl | -0.1 | -0.1 to 0 | <0.001 |
| Current smoking** | 0.1 | 0 to 0.2 | 0.12 |
|  |  |  |  |
| PLR initial diameter | 0.2 | 0.2 to 0.2 | <0.001 |
| Fentanyl | 0 | -0.1 to 0 | 0.043 |
| SOFA | 0 | 0 to 0 | 0.002 |
|  |  |  |  |
| PLR initial diameter | 0.2 | 0.2 to 0.3 | <0.001 |
| Fentanyl | 0 | -0.1 to 0 | 0.001 |
| SAPS 3 | 0 | 0 to 0 | 0.86 |
|  |  |  |  |
| PLR initial diameter | 0.2 | 0.1 to 0.2 | <0.001 |
| Fentanyl | 0 | -0.1 to 0 | 0.054 |
| Delirium*** | 0 | -0.1 to 0.2 | 0.41 |
|  |  |  |  |
| PLR initial diameter | 0.2 | 0.2 to 0.2 | <0.001 |
| Fentanyl | 0 | -0.1 to 0 | 0.011 |
| Septic shock*** | -0.1 | -0.2 to 0 | 0.087 |
|  |  |  |  |

*) Alcohol intake above recommended by Danish Health Authorities in 2020 (84g of pure alcohol for women/168g for men per week). Alcohol consumption data from 50 patients.

**) Smoking data from 50 patients

***) If in septic shock/delirium at some point throughout the ICU stay.
